# Supplementary material for: Unraveling Lifelong Brain Morphometric Dynamics: A Protocol for Systematic Review and Meta-Analysis in Healthy Neurodevelopment and Ageing
Source: Biomedicines. 2023 Jul 14;11(7):1999. doi: 10.3390/biomedicines11071999 (PMC10377186; doi:10.3390/biomedicines11071999)
Supplement: Supplementary file 1 [file biomedicines-11-01999-s001.zip › Supplementary File S2_Search_strategy_for_PubMed.pdf]

Preliminary searches on "Structural change in the brain over a lifespan: a protocol for systematic review and meta-analysis"
Source: PubMed

Search No	Search string	Results	Notes
1	(("MRI"[Title/Abstract] OR "Magnetic Resonance Imaging"[Title/Abstract]) AND (("brain"[Title/Abstract] OR "brains"[Title/Abstract] OR "brain stem"[Title/Abstract] OR "cingulate cortex"[Title/Abstract] OR "entorhinal cortex"[Title/Abstract] OR "fornix"[Title/Abstract] OR "temporal pole"[Title/Abstract] OR "3rd ventricle"[Title/Abstract] OR "4th ventricle"[Title/Abstract] OR "5th ventricle"[Title/Abstract] OR "amygdala"[Title/Abstract] OR "angular gyrus"[Title/Abstract] OR "anterior orbital gyrus"[Title/Abstract] OR "basal ganglia"[Title/Abstract] OR "basal nuclei"[Title/Abstract] OR "basolateral nucleus"[Title/Abstract] OR "calcarine sulcus"[Title/Abstract] OR "caudal anterior cingulate cortex"[Title/Abstract] OR "caudal middle frontal gyrus"[Title/Abstract] OR "caudate nucleus"[Title/Abstract] OR "central fissure"[Title/Abstract] OR "central nucleus of the amygdala"[Title/Abstract] OR "centrum medianum"[Title/Abstract] OR "cerebellar cortex"[Title/Abstract] OR "cerebral cortex"[Title/Abstract] OR "white matter"[Title/Abstract] OR "cerebrospinal fluid"[Title/Abstract] OR "choroid plexus"[Title/Abstract] OR "cingulate gyrus"[Title/Abstract] OR "gyrus cinguli"[Title/Abstract] OR "corpus callosum"[Title/Abstract] OR "cuneus"[Title/Abstract] OR "deiters's nucleus"[Title/Abstract] OR "fornicate"[Title/Abstract] OR "frontal pole"[Title/Abstract] OR "frontomarginal gyrus"[Title/Abstract] OR "fusiform gyrus"[Title/Abstract] OR "globus pallidus"[Title/Abstract] OR "gray	8,408	Filters:<br>English language<br>Publication date: 1990-2023

	matter"[Title/Abstract] OR "gyrus rectus"[Title/Abstract] OR "hippocampus"[Title/Abstract] OR "inferior frontal gyrus"[Title/Abstract] OR "inferior horn of lateral ventricle*"[Title/Abstract] OR "inferior occipital gyrus"[Title/Abstract] OR "Inferior parietal lobule"[Title/Abstract] OR "Inferior temporal gyrus"[Title/Abstract] OR "Insula"[Title/Abstract] OR "internal capsule*"[Title/Abstract] OR "lateral fissure"[Title/Abstract] OR "lateral occipital cortex"[Title/Abstract] OR "lateral occipital sulcus"[Title/Abstract] OR "lateral orbital gyrus"[Title/Abstract] OR "lateral orbitofrontal cortex"[Title/Abstract] OR "lateral temporal lobe"[Title/Abstract] OR "lateral ventricles"[Title/Abstract] OR "lingual gyrus"[Title/Abstract] OR "long gyri"[Title/Abstract] OR "medial dorsal nucleus"[Title/Abstract] OR "medial orbital gyrus"[Title/Abstract] OR "medial orbitofrontal cortex"[Title/Abstract] OR "middle frontal gyrus"[Title/Abstract] OR "middle occipital gyrus"[Title/Abstract] OR "middle temporal gyrus"[Title/Abstract] OR "nucleus accumbens"[Title/Abstract] OR "occipital pole"[Title/Abstract] OR "operculum"[Title/Abstract] OR "optic Chiasm"[Title/Abstract] OR "orbital gyrus"[Title/Abstract] OR "paracentral lobule"[Title/Abstract] OR "parahippocampal cortex"[Title/Abstract] OR "parahippocampal gyrus"[Title/Abstract] OR "paraterminal gyrus"[Title/Abstract] OR "parietal lobe"[Title/Abstract] OR "pars opercularis"[Title/Abstract] OR "pars orbitalis"[Title/Abstract] OR "pars triangularis"[Title/Abstract] OR "pericalcarine cortex"[Title/Abstract] OR "perirhinal cortex"[Title/Abstract] OR "planum temporale"[Title/Abstract] OR "postcentral gyrus"[Title/Abstract] OR "posterior cingulate cortex"[Title/Abstract] OR "posterior orbital gyrus"[Title/Abstract] OR "posterior parietal cortex"[Title/Abstract] OR "precentral gyrus"[Title/Abstract] OR "Precuneus"[Title/Abstract] OR "prepiriform		

	cortex"[Title/Abstract] OR "Putamen"[Title/Abstract] OR "rostral anterior cingulate cortex"[Title/Abstract] OR "Rostral middle frontal gyrus"[Title/Abstract] OR "short gyri"[Title/Abstract] OR "straight gyrus"[Title/Abstract] OR "subcentral gyrus"[Title/Abstract] OR "substantia nigra"[Title/Abstract] OR "superior frontal gyrus"[Title/Abstract] OR "Superior occipital gyrus"[Title/Abstract] OR "superior temporal gyrus"[Title/Abstract] OR "Supramarginal gyrus"[Title/Abstract] OR "Thalamus"[Title/Abstract] OR "Transverse temporal gyrus"[Title/Abstract] OR "ventral diencephalon"[Title/Abstract] OR "Vessels"[Title/Abstract] OR "visual cortex"[Title/Abstract] OR "Cerebellum"[Title/Abstract] OR "Cochlear Nucleus"[Title/Abstract] OR "Medulla Oblongata"[Title/Abstract] OR "Pineal gland"[Title/Abstract] OR "Hypothalamus"[Title/Abstract] OR "Cerebrum"[Title/Abstract] OR "Olfactory cortex"[Title/Abstract]) AND ("structure*"[Title/Abstract] OR "size"[Title/Abstract] OR "volume*"[Title/Abstract] OR "thick*"[Title/Abstract] OR "dimension*"[Title/Abstract] OR "organ size"[MeSH Terms]) AND ("change*"[Title/Abstract] OR "structural damage*"[Title/Abstract] OR "shrink*"[Title/Abstract] OR "Atrophy"[Title/Abstract] OR "atrophies"[Title/Abstract] OR "Atrophy"[MeSH Terms]) AND ("age"[Title/Abstract] OR "ages"[Title/Abstract] OR "Aging"[Title/Abstract] OR "lifespan*"[Title/Abstract] OR "life span*"[Title/Abstract] OR "senescence"[Title/Abstract] OR ("Aging"[MeSH Terms] OR "Age Factors"[MeSH Terms] OR "Healthy Aging"[MeSH Terms])))) AND (1990:2023[pdat])		
